# Antifungal efficacy of caffeic acid and nano-caffeic acid particles against candidiasis: an ***in vitro*** study

**DOI:** 10.1080/20002297.2025.2564690

**Published:** 2025-10-07

**Authors:** Maede Salehi, Iman Haghani, Majid Saeedi, Katayoun Morteza-Semnani, Reza Negarandeh, Abolfazl Hosseinnataj, Ali Jafari, Anahita Lotfizadeh, Anahita Rafiei, Tahereh Molania

**Affiliations:** a Department of Oral Medicine, Dental Research Center, Mazandaran University of Medical Sciences, Sari, Iran; b Faculty of Dentistry, Mazandaran University of Medical Sciences, Sari, Iran; c Invasive Fungi Research Center, Communicable Diseases Institute, Mazandaran University of Medical Sciences, Sari, Iran; d Department of Medical Mycology, School of Medicine, Mazandaran University of Medical Sciences, Sari, Iran; e Department of Pharmaceutics, Faculty of Pharmacy, Mazandaran University of Medical Sciences, Sari, Iran; f Department of Medicinal Chemistry, Faculty of Pharmacy, Mazandaran University of Medical Sciences, Sari, Iran; g Pharmaceutical Sciences Research Center, Health Research Institute, Babol University of Medical Sciences, Babol, Iran; h Department of Biostatistics, Faculty of Health, Mazandaran University of Medical Sciences, Sari, Iran; i Dentist, Sari, Iran

**Keywords:** Antifungal activity, caffeic acid, *Candida* species, candidiasis, nano-caffeic acid

## Abstract

**Background/purpose:**

Candidiasis is the most common oral fungal infection. Several medications have been introduced to manage this infection. This study investigated the antifungal effect of caffeic acid and nano-caffeic acid.

**Materials and methods:**

The size and particle dispersion index (PDI) of caffeic acid-containing niosome vesicles were measured after their production. The zeta potential was measured using a Zetasizer Nano ZS, and the amount of nano-caffeic acid released from the vesicles was measured. *Candida* isolates were cultured in Malt Extract Agar medium. Nystatin, fluconazole, caffeic acid and nano-caffeic acid were studied according to the Clinical and Laboratory Standards Institute (CLSI) protocol (M27-A3/S4), a broth microdilution test was performed, and the minimum inhibitory concentration (MIC) was determined. The data were analyzed using the Mann‒Whitney and Kruskal‒Wallis tests.

**Results:**

The optimal formulation had 100 mg Tween 60, 100 mg Span 60, 200 mg cholesterol, a size of 271.83 ± 3.11 nm, a PDI of 0.21 ± 0.02, a zeta potential of 5.58 ± 0.47 mV and an encapsulation efficiency (EE%) of 42.34 ± 4.34%. The size, absolute zeta potential and EE% increased significantly with increasing cholesterol content from zero to 200  mg (*P *< 0.05). Caffeic acid, nano-caffeic acid, carrier, fluconazole and nystatin had the lowest to highest antifungal activity, respectively.

**Conclusion:**

According to the MIC_50_ and MIC_90_ values, nystatin, fluconazole, carrier, nano-caffeic acid and caffeic acid had the highest to lowest inhibitory efficiency against *Candida* species, respectively.

## Introduction

*Candida* can be found in the mucous membrane of the skin, genitals, nose, mouth, ears and eyes as a harmless fungus and an organism in normal flora [1]. *Candida* species are present in the oral cavity of 54−71.4% of healthy individuals [2]. In immunocompromised patients such as infants, the elderly wearing dentures, patients with AIDS, patients taking broad-spectrum antibiotics, immunosuppressive and anti-cancer drugs, and people with oral mucosa breakdown and decreased saliva, the balance of the body's normal flora is disturbed; thus, the risk of *Candida* infection, which is called candidiasis, increases [1,3–9]. The fourth most frequent cause of nosocomial blood-stream infections is *Candida* species [10].

Common antifungal drugs used in the treatment of *Candida*, in addition to their benefits, have limitations such as drug interactions, and the reduced sensitivity of fungal species to these drugs, including nystatin and azole derivatives, is another major complication. Therefore, alternative and new treatment options should be investigated [1,11] Currently, scientists pay special attention to herbal medicines because of their relatively few adverse effects [12]. Additionally, herbal medicines and natural substances derived from them have been effectively used in dentistry [13–16]. Previous studies have reported the antifungal efficacy of several natural substances, including tea tree oil, manuka honey and manuka oil [17]. Natural substances exert their antifungal effects by weakening the cell walls and inhibiting ergosterol production, and disrupting the integrity of the cell membrane [18].

All plant species can produce the phenolic chemical caffeic acid, which is found in drinks such as tea and coffee. The antioxidant, anti-inflammatory, anti-cancer, antibacterial, antiviral and wound-healing properties of this phenolic acid and its derivatives are demonstrated [19–23]. The anticancer effects of caffeic acid phenethyl ester (CAPE) are mediated through the inhibition of matrix metalloproteases (MMPs) 2 and 9 and nuclear factor kappa B [24] Additionally, in studies, the antioxidant and anti-inflammatory activities of CAPE, including inhibiting the release of arachidonic acid from the cell membrane, suppressing T cells, and modulating the immune system, have been observed [25,26]. Recently, in several studies, the antifungal activity of CAPE against *C. albicans* has been reported; this substance has prevented the growth of *C. albicans* and biofilm formation [27–29]. De Vita et al. assessed the antifungal effects of caffeic acid and its esters against *C. albicans* biofilms, focusing on their ability to inhibit biofilm formation or disrupt existing biofilms. The compounds were tested at various stages: early (4 h) and mature (24 h), with fluconazole as a reference. The findings revealed that caffeic acid ester 7, cinnamic acid ester 8, and 3,4-dihydroxybenzoic acid ester 10 had significantly greater antibiofilm activity than fluconazole. This study also highlighted that these compounds possess activity against planktonic *C. albicans* cells, albeit to a lesser extent than fluconazole, suggesting that their primary mechanism involves interference with biofilm adhesion and aggregation processes [30]. In another study, Rossatto et al. investigated the antifungal and anti-virulence properties of two plant-derived polyphenols, ellagic acid and CAPE, against drug-resistant *Candida auris*. They found that ellagic acid demonstrated potent antifungal activity at low concentrations, whereas CAPE showed antifungal activity at comparatively higher concentrations. Time-kill assays showed that CAPE reduced *C. auris* cells within 4 h, outperforming fluconazole. Both compounds disrupted fungal cell wall integrity, reduced biofilm biomass and metabolic activity, and impaired adhesion to human epithelial cells. *In vivo*, they significantly prolonged the survival of *Galleria mellonella* larvae infected with *C. auris*, and ellagic acid extended the survival of *Caenorhabditis elegans* infected with *C. albicans*. CAPE did not affect survival in *C. albicans*-infected nematodes. Overall, these results underscore the potential of ellagic acid and CAPE as candidates for new antifungal therapies for resistant fungal infections [31]. Alfarrayeh et al. studied the effects of CAPE on various *Candida* species, revealing strain- and dose-dependent inhibition of growth and biofilm formation. CAPE also induced caspase-dependent and caspase-independent apoptosis in certain strains, indicating its potential as an effective inhibitor of both viability and biofilm formation in *Candida* [29]. Additionally, a murine model study investigated the antifungal and immunomodulatory effects of CAPE against *C. albicans* in oral candidiasis. CAPE showed significant antifungal activity against various strains, including fluconazole-resistant isolates. *In vitro*, it reduced biofilm biomass and viable cell counts while downregulating virulence genes linked to adhesion and invasion. *In vivo*, CAPE prolonged survival in *Galleria mellonella*, increased hemocyte counts, and enhanced antifungal peptide expression. In a murine model, it reduced the fungal load; decreased lesions, hyphal invasion, inflammation and upregulated *β*-defensin 3 expression [32].

Nanotechnology is a modern science that is frequently applied to medical procedures [33]. Achieving cost-effective synthesis and producing nanoparticles or nanocomposites smaller than 100 nm are essential for the burgeoning use of nanotechnology [34]. Studies have shown that materials, products, and devices made of nanoparticles have different properties from larger particles; including a rapid response rate, increased efficiency, functional effectiveness and decreased drug side effects [35,36]. Various nanostructures have been applied in dentistry and medicine and have improved the systematic and oral health of patients [37]. Previous studies have demonstrated the great efficacy of nanostructures against *C. auris*, including Ag–Cu–Co trimetallic nanoparticles and nitric oxide nanoparticles [38]. A study conducted by Shahin et al. showed the effectiveness of nano-liposomal formulation of caffeic acid in healing acute pancreatitis in rats. This alleviation primarily occurs through the anti-oxidant, anti-inflammatory and anti-apoptotic properties of this substance [39].

The purpose of this study is the production of nano-caffeic acid using nanoscience and the use of other tools and facilities to produce this substance better and with higher quality and to have the most significant impact on candidiasis. Additionally, in this study, caffeic acid and nano-caffeic acid were compared in terms of their effectiveness at different concentrations.

## Materials and methods

### Materials

Caffeic acid (CA) (Sigma-Aldrich, USA), Span 60, tween 60 and cholesterol (Chol.) were acquired from Merck (a pharmaceutical company based in Germany), Samchun (Korean Pure Chemical Co., Ltd.), Sharlua (Sharlab S.L. in Spain) and Merck, respectively. The process of purifying distilled water involved utilizing a Human Power 2 device manufactured by Human Co. in Korea. Additionally, the source of the ethanol used was Merck.

### Fabrication of caffeic acid noisome (caffeosome)

The noisome vesicles were prepared using modified ethanol injection processing [33]. Initially, a mixture of cholesterol, CA, span 60 and ethanol (forming an oily phase) was prepared in a beaker using magnetic stirring at a speed of 1500 rpm (rounds per minute). The mixture was heated to a temperature range of 75–80°C until complete dissolution occurred. The specific composition details for each formulation can be found in Table 1. Next, the aqueous phase, a mixture of Tween 60 and water, was transferred to a separate beaker. This mixture was placed on a hot plate until it reached the same temperature as the oily phase. Subsequently, the oily phase is injected into the aqueous phase using a syringe. The mixture was placed on the heater stirrer for 10 min, maintaining the same temperature and rotation speed. The beaker's content was homogenized for 15 min at a speed of 9000 rpm using a homogenizer in a warm water bath. Subsequently, a probe sonicator (Bandelin 3100; Germany) was employed to subject the MLV niosome to sonication at a 20% amplitude for 3 min. This process aimed to generate smaller caffeic acid-loaded niosomes before freezing the combination in an ice bath.

**Table 1.** Ingredients and nanovesicle characteristics. The data consists of the mean and standard deviation of three different classifications.

Formulation	CA (mg)	Tween 60 (mg)	Span 60 (mg)	Chol. (mg)	Ethanol (ml)	Water (ml)	Size (nm)	PDI	Zeta *P*. (mVolt.)	EE (%)
Caffeosome 1	25	100	100	0	3	Up to 17	167.067 ± 8.252	0.238 ± 0.003	−3.8 ± 0.147	16.78 ± 1.837
Caffeosome 2	25	100	100	50	3	Up to 17	187.9 ± 5.381	0.183 ± 0.007	−5.267 ± 0.375	18.453 ± 1.27
Caffeosome 3	25	100	100	100	3	Up to 17	230.7 ± 6.236	0.143 ± 0.012	−5.317 ± 0.404	21.55 ± 0.981
Caffeosome 4	25	100	100	150	3	Up to 17	246.5 ± 8.218	0.142 ± 0.008	−5.33 ± 0.517	24.64 ± 2.595
Caffeosome 5	25	100	100	200	3	Up to 17	271.833 ± 3.107	0.208 ± 0.015	−5.58 ± 0.471	42.337 ± 4.336

### Characterization of noisome vesicle

The diameter and polydispersity index (PDI) of the niosome vesicles were determined at a temperature of 25 °C using two different instruments: the dynamic light scattering (DLS) Mastersizer 2000 from Malvern Panalytical Technologies in the UK and the Zetasizer Nano ZS system from Malvern Instruments, Worcestershire, in the UK. We employed Laser Doppler electrophoresis to determine the zeta potential of the malodorous vesicles [40]. Three specimens were collected for each formulation, with the specific details of each caffeosome formulation provided in Table 1.

### Encapsulation efficiency (EE%) measurement

We utilized the centrifugation technique to determine the proportion of CA that was encapsulated within the niosomal vesicles. The colloidal specimens were subjected to centrifugation at a speed of 19,000 revolutions per minute using a SIGMA centrifuge model 3−30 KS from Germany for 45 min. Subsequently, the supernatant was filtered through a 0.22 μm pore size membrane. The quantity of unbound CA in the filtered solution was determined using a UV spectrophotometer set at a wavelength of 311 nm (UV Vis JASCO V-630, UK) [41]. The percentage of each formulation's EE is provided in Table 1.

_$\mathrm{EE}\%\;=\;(W_{\mathrm{initial}}–W_{\mathrm{free}})/W_{\mathrm{initial}}$_

W_free_ represents the quantity of drug present in the supernatant, while W_initial_ represents the quantity of drug added to the formulation.

### Morphology analysis

We employed field emission scanning electron microscopy (FE-SEM) to assess the shape of noisome vesicles. A small amount of the specimen was applied to a copper grid covered with a layer of carbon. Subsequently, the sample was desiccated using ambient air and subjected to sputter coating with gold to increase its conductivity. The images were obtained using a scanning electron microscope (HITACHI S-4160) at an acceleration voltage of 20 kilovolts and a magnification of 15,000 times [42].

### ATR-FTIR spectroscopy

The interaction between caffeic acid and the other excipients was assessed using a Cary 630 FTIR spectrophotometer (Agilent Technologies Inc., CA, United States) equipped with a diamond ATR. The ATR-FTIR tests were conducted on caffeosome powder (a freeze-dried formulation), CA, chol., Tween 60 and Span 60. The ATR-FTIR spectra were recorded at room temperature within the frequency range of 4000–400 cm^−1^, with a resolution of 2 cm^−1^ [43,44].

#### In vitro drug release

The release was identified using Dissolution Apparatus II and Immersion Cells equipped with an acetate cellulose membrane (MWCO 12 kDa). The specimens were inserted into the compartments and enclosed by an acetate cellulose membrane [45]. Subsequently, the cells were immersed in a dissolution medium consisting of 150 mL of water:ethanol (90:10) solution in each beaker. The temperature was maintained at 37 °C while the beakers were rotated at a speed of 100 rpm. At various time points (1, 2, 4, 6, 8 and 24 h), 5 ml of the dissolving medium was extracted and filtered through 0.22 μm filter paper. In addition, we introduced 5 mL of water:ethanol (90:10) into the dissolving medium of each beaker after each sampling to maintain the volume of the dissolution medium. The concentration of CA was determined at a wavelength of 311 nm using a UV spectrophotometer.

### Preparation of culture medium and Candida isolates

In this experimental-laboratory study, 20 *Candida* isolates, including 15 *C. albicans* isolates, 3 *C. krusei* isolates and 2 *C. glabrata* isolates taken from clinical samples were cultured in Malt Extract Agar (MEA) medium and prepared for the study. All the samples were previously identified by molecular methods, such as sequencing based on the ITS1-5.8S-ITS gene region and PCR-RFLP and sequencing methods.

### Antifungal susceptibility testing

Antifungal susceptibility testing was performed using broth microdilution methods as outlined in the Clinical and Laboratory Standards Institute (CLSI) M27-A3/S4 document, which uses RPMI 1640 medium (Sigma Chemical Co.) buffered to pH 7.0 with 0.165 M morpholinepropanesulfonic acid (MOPS) (Sigma) with L-glutamine and no bicarbonate. The fluconazole (Sigma), nystatin (Sigma) and caffeic acid (Sigma–Aldrich, USA) stock solutions were prepared in DMSO (dimethylsulfoxide), and the final concentrations of antifungal drugs in the wells ranged from 0.125 to 128 μg/ml caffeic acid and nano caffeic acid, 0.016–16 μg/ml for nystatin, and the carrier and 0.063–64 μg/ml for fluconazole. *Candida* suspensions were prepared from colonies cultured on malt extract agar (MEA, Difco Laboratories, Detroit, MI, USA) at 35 °C. The suspensions were adjusted spectrophotometrically to achieve 75−77% transmission at a wavelength of 530 nm. They were then diluted in RPMI 1640 medium to obtain final inocula ranging from 0.5 to 5 × 10^3^ cells/ml. The microdilution plates were incubated at 35 °C for 24−48 h. The MIC was determined visually as the lowest concentration of each drug that resulted in at least 50% growth inhibition, except for nystatin. For nystatin, the MIC was defined as the lowest concentration that achieved 100% growth inhibition. Owing to quality control challenges, *Candida krusei* (ATCC 6258) and *Candida parapsilosis* (ATCC 22019) were used, with all antifungal susceptibility tests performed in duplicate.

### Statistical analysis

In this study, descriptive indices such as the means, standard deviations and quartiles were used. The normality of the data was evaluated using the Shapiro‒Wilk test, and the results showed that *C. albicans* did not follow this hypothesis; thus, non-parametric tests were used. Finally, Mann‒Whitney and Kruskal‒Wallis tests were used. All tests were performed at a significance level of 0.05 using Graphpad Prism 6 and SPSS 22.

## Results

We assessed the hydrodynamic diameter and PDI of the nanoparticles (NPs) using DLS to confirm the formation of nano vesicles and the quality of the particle size distribution width. The magnitude of the zeta potential significantly affects the stability of the nanovesicles. Different concentrations of cholesterol (0, 0.25, 0.5, 0.75 and 1 w/w) were assessed to optimize the niosomal formulations of CA. Table 1 displays the components and characteristics of the niosomal vesicles.

The optimized nanovesicle, known as Caffeosome 5, was produced using the ethanol injection approach. It has the following properties: a particle size of 271.83 ± 3.11 nm, a PDI of 0.208 ± 0.01, a zeta potential of −5.58 ± 0.47 mV, and a drug encapsulation percentage of 42.34 ± 4.37%.

The results of our study demonstrated that the size of the vesicle expanded as the amount of cholesterol increased (*P* < 0.001). The PDIs ranged from 0.142 to 0.238 (Table 1), indicating that the noisome vesicles were reasonably uniform in size.

The zeta potential values at a temperature of 25 °C ranged from −3.8 to −5.58 mV (Table 1). These values indicate the presence of a repulsive electrostatic force between the vesicles, leading to the formation of stable formulations. Our investigation revealed that increasing the cholesterol content in formulations from 0 to 1 **%** w/w resulted in a significant increase in the absolute zeta potential of noisome vesicles (*P* < 0.001).

The percentage of encapsulated CA in all formulations varied from 16.78 to 42.34 (Table 1). When the amount of cholesterol in caffeosome 5 was 200 mg (1% w/w), the greatest drug encapsulation efficiency was observed compared with that of the other formulations. When the concentration of Chol. When the concentration increased from 0 to 1% w/w, the encapsulation efficacy increased significantly (*P *< 0.001). The CA is a lipophilic molecule that can be placed in the hydrophobic bilayers. The characterization investigations were exclusively conducted for the caffeosome 5 formulations (Table 1).

**Figure 1.**
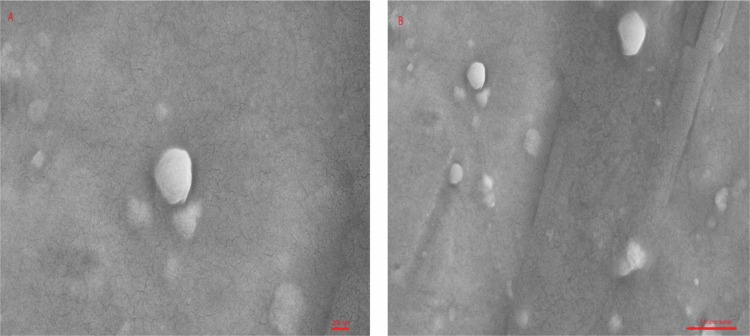
Morphology of caffeosome 5.

This method can be used to determine the diameter and surface features of the vesicle. The caffeosome 5 exhibits a semi-spherical shape (Figure 1A) and displays a well-distributed range of sizes (Figure 1B). These results align with the results acquired from the DLS technique described in the preceding sections of this investigation.

**Figure 2.**
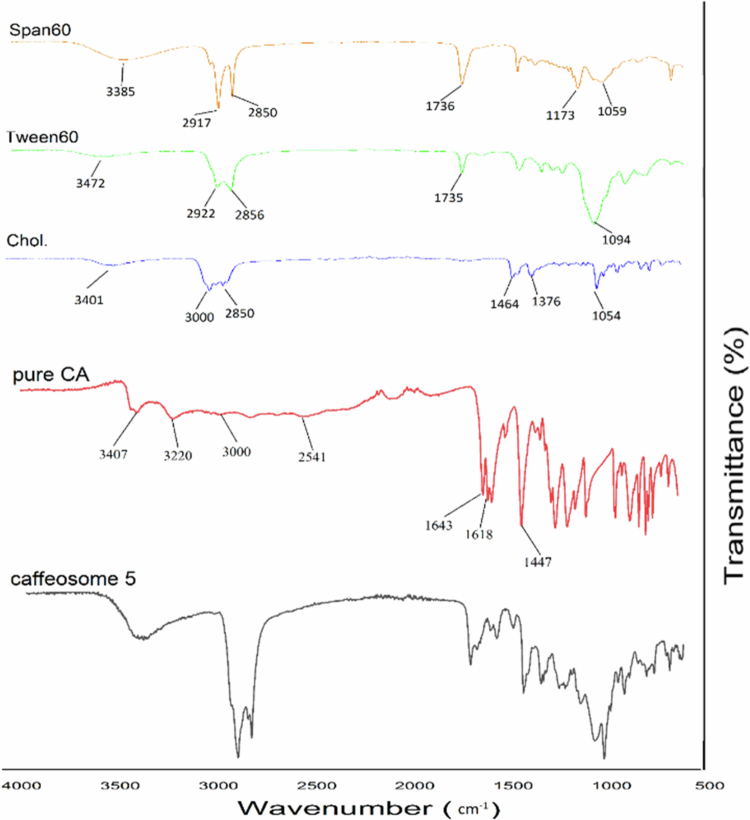
ATR-FTIR spectra of caffeosome, pure CA, Chol., Tween60 and Span60.

Figure 2 displays the ATR-FTIR spectra of freeze-dried niosome powder, CA, cholesterol, Tween 60, and Span 60. The ATR-FTIR spectrum of pure CA exhibited distinct peaks at specific wavenumbers, namely, 3407 and 3220 cm^−1^ (indicating the stretching of phenolic O─H groups), 3150−3000 cm^−1^ (representing the stretching of aromatic C─H and alkenyl C─H groups), 2541 cm^−1^ (indicating the stretching of the O─H group in the carboxylic group), 1643 cm^−1^ (representing the stretching of the C═O bond), and 1618−1447 cm^−1^ (indicating the stretching of alkenyl C═C and aromatic C═C bonds). The cholesterol spectrum exhibited distinct peaks at specific wavenumbers: 3401 cm^−1^ (representing O─H stretching), 3000−2850 cm^−1^ (representing asymmetric and symmetric stretching of C─H bonds in CH2 and CH3 groups), 1464−1376 cm^−1^ (representing bending of C─H bonds), and 1054 cm^−1^ (representing C─O stretching). Tween 60 has prominent peaks at 3472 cm^−1^ (corresponding to O─H stretching), 2922 cm^−1^ (representing C─H asymmetric stretching), 2856 cm^−1^ (indicating C─H symmetric stretching), 1735 cm^−1^ (associated with C═O stretching), and 1094 cm^−1^ (reflecting C─O stretching). Span 60 exhibited prominent peaks at 3385 cm^−1^ (corresponding to O─H stretching), 2917 cm^−1^ (representing C─H asymmetric stretching), 2850 cm^−1^ (indicating C─H symmetric stretching), 1736 cm^−1^ (associated with C═O stretching) and 1173 and 1059 cm^−1^ (related to C─O stretching). The ATR-FTIR results indicate that there was no chemical interaction observed between CA and the excipients in the caffeosome 5.

**Figure 3.**
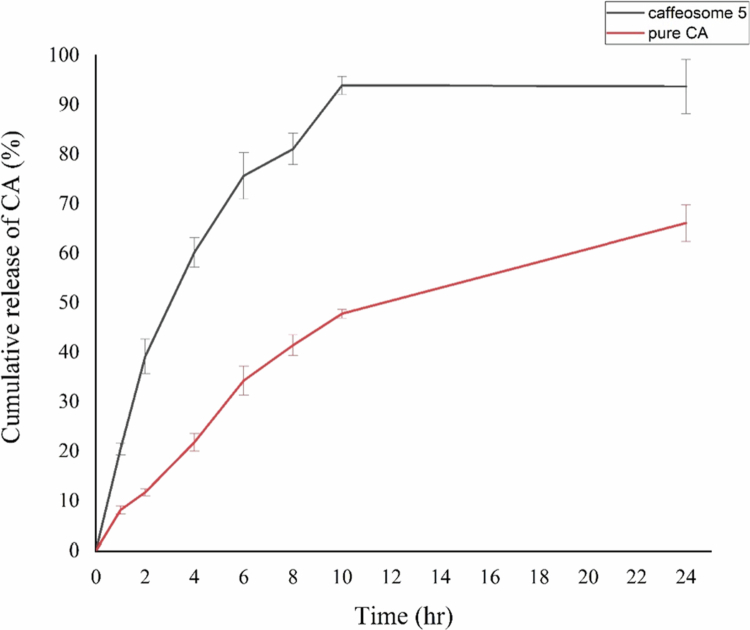
Dissolution profile of plain CA solution and caffeosome 5 formulation.

Figure 3 displays the drug release profiles of the caffeosome and pure CA in an *in vitro* setting. The Higuchi release model demonstrated the highest accuracy (*R*^2^ = 0.851) for the caffeosome formulation. The first-order, zero, Korsmeyer‒Peppas, and Hixon‒Crowell kinetic models yielded *R*^2^ values of 0.74, 0.579, 0.565 and 0.353, respectively. The study revealed that after 24 h, the CA was released faster from the caffeosome 5 formulation (93.71 ± 5.46) than from the pure CA formulation (66.12 ± 3.66) (*P *< 0.001).

An accelerated study of the optimal nanovesicles was conducted over three months at two different temperatures (room and refrigerator temperatures) in a tightly sealed container. This study investigated how temperature and time influence important factors, including average particle size (z-ave), PDI, zeta potential, encapsulation efficacy (EE%), and the formation of clumps. In this regard, no significant changes were observed in the mean particle size, PDI, absolute zeta potential, or EE% during the first month, either at room or refrigerator temperatures (*P*-value > 0.05). Also, all the parameters indicate that no change was observed in the nanoparticles after three months at the refrigerator temperature. However, after three months, the mean size of the nanoparticles significantly increased at room temperature (*P*-value < 0.05), and both the absolute zeta potential and EE% significantly decreased (*P*-value < 0.05), but the PDI value did not change significantly (*P*-value > 0.05). The color of the nanoparticles was milky throughout the study, and no precipitation was observed.

Table 2 displays the MIC _50_, MIC _90_, geometric mean MIC values, mode MIC values and MIC range distributions of the tested antifungal agents. Caffeic acid, nanocaffeic acid, the carrier and two comparator antifungals (fluconazole and nystatin) were tested against all *Candida* species. Caffeic acid had MIC values ranging from 64 to 128  μg/mL against all *Candida* strains, compared with 4−128 μg/mL for nanocaffeic acid, 2−4 μg/mL for the carrier, 0.063−1 μg/mL for fluconazole and 0.032−1 μg/mL for nystatin. The MIC values for each *Candida* species are presented in Table 2. Notably, *C. albicans* isolates presented relatively high MICs for caffeic acid (mostly 128 µg/mL), while *C. tropicalis* and *C. glabrata* showed relatively low MICs in several cases. These findings suggest species-specific variability in susceptibility to caffeic acid and its nano formulation. There were significant differences between the MICs of caffeic acid and nano-caffeic acid with fluconazole (*P* < 0.001) and with nystatin (*P* < 0.001). Notably, the results showed that the difference in the geometric mean MIC values of caffeic acid and the other three antifungal agents (nano-caffeic acid, fluconazole and nystatin) was statistically significant (*P *< 0.001). In general, based on GM MIC and MIC_50_, the results indicated that nano-caffeic acid was much more effective on *Candida* species compared to caffeic acid. Table 3 shows the Spearman's correlation coefficient results between groups (Table 3).

**Table 2.** *In vitro* susceptibility of 20 *Candida* species isolated from oral candidiasis against 5 antifungal agents.

	Antifungal agents MICs (μg/ml)				
*Candida* species	Caffeic acid	Nano caffeic acid	Fluconazole	Nystatin	Carrier
*C. albicans*	128	4	0.063	0.5	2
*C. albicans*	128	16	0.25	0.25	2
*C. albicans*	128	4	1	0.25	4
*C. albicans*	128	2	0.125	0.063	2
*C. albicans*	64	4	1	0.125	4
*C. albicans*	128	4	0.125	0.5	4
*C. albicans*	128	32	1	0.063	4
*C. albicans*	128	16	0.125	0.063	2
*C. albicans*	128	16	0.125	0.063	4
*C. albicans*	128	4	0.25	0.125	4
*C. albicans*	128	4	0.5	0.5	2

MIC parameter (μg/ml) of antifungal agents against *C. albicans*					
MIC_50_	128	4	0.25	0.125	4
MIC_90_	128	16	1	0.5	4
G-mean	120.1831566	6.622026239	0.26645322	0.1612998	2.918960211
Mode	128	4	0.125	0.063	4
MIC range	64−128	2−32	0.063−1	0.063−0.5	2−4

MIC (μg/ml) of antifungal agents against other *Candida* species					
*C. krusei*	64	16	0.125	1	4
*C. krusei*	128	4	0.125	0.063	4
*C. krusei*	128	128	1	0.032	2
*C. glabrata*	128	32	0.5	1	4
*C. glabrata*	64	4	1	0.125	2
*C. glabrata*	128	8	0.5	0.125	4
*C. tropicalis*	64	4	0.063	0.5	2
*C. tropicalis*	128	16	1	0.125	2
*C. tropicalis*	128	4	0.5	0.25	2

MIC parameter (μg/ml) of antifungal agents against all species (*n* = 20)					
MIC_50_	128	4	0.25	0.125	4
MIC_90_	128	32	1	0.5	4
G-mean	111.4304721	8.28211939	0.30803145	0.177339362	2.82842
Mode	128	4	1	0.063	2
MIC range	64−128	4−128	0.063−1	0.032−1	2−4

**Table 3.** Spearman's correlation coefficient between groups.

Group	Caffeic acid	Nano caffeic acid	Carrier	Fluconazole	Nystatin
Caffeic acid	–	−0.21	−0.19	−0.14	−0.30
Nano caffeic acid		–	−0.22	0.14	0.14
Carrier			–	0.02	−0.18
Fluconazole				–	−0.24
Nystatin					–

Table 4 demonstrated the effect size between groups (Table 4). Owing to the failure to establish the normality hypothesis and large values ​​for the standard deviation, confidence intervals were not reported.

**Table 4.** The effect size between groups.

Group	Caffeic acid	Nano caffeic acid	Carrier	Fluconazole	Nystatin
Caffeic acid	–	2.46	2.51	2.85	2.88
Nano caffeic acid		–	0.41	4.07	4.47
Carrier			–	3.25	3.58
Fluconazole				–	0.52
Nystatin					–

*A value greater than 0.8 indicates a strong effect size between the two groups.

## Discussion

The majority of opportunistic fungal pathogens that cause fungal infections in patients with impaired or compromised immune systems are thought to be *Candida* species [7]. *Candida* species have shown resistance to Azole derivatives due to their general and long-term use [8]. Thus, new medications should be implemented. In this study, we investigated caffeic acid, a substance made by various species, because of its medicinal properties. Furthermore, in the present study, the nanoform of this substance was produced and compared with caffeic acid in terms of its effectiveness.

Ideal nanoparticles are those with a smaller particle size, narrower distribution, high zeta potential, and high EE%. The findings of our investigation demonstrated that the size of the vesicle expanded as the amount of cholesterol increased (*P* < 0.001). In line with our study, Abootorabi et al. found that elevated cholesterol levels in niosomes can lead to an increase in niosome vesicle diameter [41]. Furthermore, Ghardashpour et al. examined the correlation between cholesterol content and vesicle particle size. They demonstrated that increasing the cholesterol concentration from 0 to 1% w/w resulted in the formation of larger vesicles [33]. The value of PDI can range from 0 to 1. A value closer to zero shows an excellent homogenous dispersion [41]. In the present study, a PDI ranging from 0.142 to 0.238 was obtained, indicating that the vesicles were homogenous in size [43].

The absorption of the ionic surfactant layer's absorption or the nature of the particle or vesicle's surface in colloidal systems results in an electric charge on the surface, which is dependent on several factors, including the kind of surface-active component utilized. The vesicle surface and surrounding factors (pH, temperature, and ionic strength) affect the surfactant concentration [36]. The zeta potential of a vesicle plays an essential role in niosome features. The surface charge of lipid systems is an important factors in the stability of nanocarriers in such a way that as the surface charge of the lipid system increases, the amount of repulsive force between the constituent particles of the system increases, and the possibility of accumulation and precipitation of nanoparticles decreases; as a result, the stability of the lipid system increases. [46] In the present study, the zeta potential of all nanocarrier niosome formulations containing caffeic acid was negative.

Our investigation revealed that increasing the cholesterol content in formulations from 0 to 1% w/w resulted in a significant increase in the absolute zeta potential of noisome vesicles (*P* < 0.001). Tajbakhsh et al. demonstrated that the absolute zeta potential of the testosome (testosterone enanthate loaded in nanovesicles) increased with the addition of increasing amounts of cholesterol in the nanovesicle formulation [43]. Abootorabi et al. reported that by increasing the cholesterol content in noisome vesicles from 0.25 to 1.25% w/w, the absolute zeta potential decreased, but this change was not significant [41]. Ghardashpour et al.'s study indicated that increasing the cholesterol content of cinnosome particles from 0 to 1% w/w significantly decreased the absolute zeta potential of niosome vesicles [33].

Cholesterol affects the membrane permeability and EE%, which results in alterations in niosome permeability. EE% can be optimized by increasing the cholesterol content [41]. According to Tajbakhsh et al., the formula with the highest cholesterol content improved the encapsulation efficiency [43]. Similar to our investigation, the encapsulation efficacy increased drastically (*P* < 0.001) with increasing concentrations of Chol. increased from 0 to 1% w/w. Since the CA molecule is lipophilic, it can be positioned within hydrophobic bilayers. However, in a different study by Ghardashpour et al., when the concentration of chol. increased from 0.25 to 1% w/w [33], the percentage of encapsulated cinnamaldehyde decreased in noisome vesicles. This difference in results compared with our study could be due to the nature of the active pharmaceutical ingredients.

In the present study, the inhibitory activities of caffeic acid/nano caffeic acid, a carrier and two common antifungal drugs, were tested against 20 clinical *Candida* strains. All paired comparisons among the five groups revealed statistically significant differences, except for the difference between fluconazole and nystatin (*p*-value = 0.092). Additionally, in this study, according to the MIC_50_ and MIC_90_ values, nystatin, fluconazole, carrier, nano-caffeic acid, and caffeic acid had the highest to the lowest inhibitory activities against *Candida* species. According to Alfarayeh et al., caffeic acid has a strong inhibitory effect on planktonic growth and biofilm formation. It can also partially remove established biofilms from a variety of *Candida* strains. In this study, the MIC values of various caffeic acid formulations against *C. albicans* ATCC 90028 ranged from 7.81 to 125 µg/mL [29]. These outcomes are consistent with the findings of de Barros et al., who reported that CAPE may inhibit the growth of both fluconazole-resistant/sensitive strains of *C. albicans*, the MIC values for these strains ranged from 16 to >64 µg/mL [32]. A different study conducted by Possamai Rossatto et al. revealed similar findings, showing a MIC value of 8 µg/mL for *C. glabrata* and 64 µg/mL for *C. krusei*. In this study, caffeic acid effectively suppressed the growth of both fungi. Furthermore, this study demonstrated that CAPE reduced *C. auris* growth, with MIC values ranging from 1 to 64 µg/mL [31]. The results of Alfarayeh et al. showed that CAPE may enter *Candida* species cells quickly [29]. Cigut et al. observed similar outcomes while studying *Saccharomyces cerevisiae* yeast. Among the four chemicals analyzed (*p*-coumaric acid, ferulic acid, caffeic acid, and CAPE), only CAPE was found to penetrate *S. cerevisiae* cells [47]. In all the aforementioned studies, the ability of caffeic acid and CAPE to inhibit the growth of different *Candida* strains was observed. In line with these studies, the results of the present study showed that the investigated medicinal groups of caffeic acid, with MIC values equal to 8−32 µg/mL, and nano-caffeic acid, with MIC values equal to 2−4 µg/mL, inhibited the growth of *Candida* strains. The reported MIC values in these studies varied due to differences in the production methods of the medicinal products, the diverse formulations, and the fungal strains analyzed. However, the results of these studies showed that caffeic acid can effectively inhibit the growth of different *Candida* strains. Contrary to the above studies, Lima et al. found that caffeic acid is not effective at *C. albicans* and *C. tropicalis* (with IC_50_ values greater than 1000  µg/mL); however, when combined with fluconazole, it shows a synergistic effect and can be effective on both *Candida* species (IC_50_ values equal to 68.00 and 109.12 µg/mL, respectively) [48]. The contrast between the results of this study and the present study may be due to the difference in the investigated medicinal formulations and fungal strains. The observed variation in MIC values across different isolates is likely attributed to intrinsic differences among *Candida* species and possibly individual strain characteristics, such as membrane composition, efflux pump activity, or biofilm formation capacity. These biological differences are consistent with previous findings on antifungal susceptibility and contribute to the observed MIC range [49].

Phenolic substances, such as CAPE, are efficient iron absorption inhibitors. The results of the investigation by Sun et al. suggested that intracellular iron depletion may be a component of the antifungal mechanism of CAPE because of its ability to form insoluble complexes with iron ions that hinder the ability of cells to absorb iron [26]. Breger et al. reported in a study on strains of *C. albicans* that CAPE can suppress the filaments of this fungus [10]. According to other studies, CAPE may have antifungal effects on RNA, DNA, and cellular proteins, which could be targets of this compound [20]. However, Chang et al. hypothesized that the cytotoxicity and antitumor effects of CAPE might be related to the mechanism that causes cell apoptosis [50]. According to Marin et al., CAPE activates genes linked to the oxidative stress response and apoptosis. According to their findings, some polyphenolic substances may possess prooxidant activity, which can potentially cause oxidative stress in cells by either suppressing the system's antioxidants or generating large amounts of reactive oxygen species [21]. Six *Candida* strains, including *C. tropicalis* SZMC, C. *albicans* SZMC 1423, *C. albicans* ATCC 44829, *C. albicans* SZMC 1424, *C. parapsilosis* SZMC 8007, and *C. tropicalis* SZMC 1512, were found to be susceptible to apoptosis by Alfarayeh et al.'s investigation. A lack of apoptotic cell death was noted in the strains of *C. parapsilosis* SZMC 8007, *C. glabrata* SZMC 1378, and *C. glabrata* SZMC 1374, suggesting that distinct species of *Candida* and even variants within the same species can exhibit different cell death responses compared to CAPE. Furthermore, TEM images of *Candida* cells treated with CAPE revealed characteristic apoptotic signals in most *Candida* species, which slightly varied across various *Candida* species [29].

The processes of CAPE-induced apoptosis in *Candida* species were studied by Alfarayeh et al. when a broad-spectrum Pan-caspase inhibitor was used, the amount of CAPE-induced apoptosis at *C. albicans* ATCC 44829, *C. tropicalis* SZMC1366, *C. albicans* SZMC 1424, and *C. tropicalis* SZMC 1512 was significantly decreased. These findings corroborate the theory that CAPE can trigger apoptotic cell death in *Candida* by various mechanisms, in addition to measuring species- and strain-dependent cell death responses [29]. In mitochondria, CAPE induces depolarization because it can increase the permeability of the plasma membrane to ions [22]. This causes the cytosol to become filled with proapoptotic substances such as cytochrome C. This, in turn, activates the yeast metacaspase Yca1p, which in turn triggers the caspase cascade and causes apoptosis [6]. Zielińska et al. examined the potential of caffeic acid, at appropriate doses, to regulate the processes associated with intestinal inflammation. The effects of caffeic acid (10–50 μM) on the biosynthesis of cytokines and chemokines (IL-8, IL-6, and monocyte-attracting protein-1) and the production of COX-2 and PGE2 were examined in this study in human colon myofibroblasts treated with IL-1β. Additionally, research has focused on the potential of caffeic acid to chelate, reduce, and inhibit the activity of angiotensin-converting enzyme (ACE) and prevent the production of advanced glycation end products (AGEs). Owing to its intense chelating activity, caffeic acid suppresses the production of AGE and targets COX-2 and its product, PGE2, as well as IL-8 production in cells [23].

Our study showed the greater antifungal efficacy of nystatin and fluconazole compared to nano-caffeic acid. However, because nano-caffeic acid is an herbal resource with fewer side effects and because transferring substances to their nano-form develops their therapeutic properties, this substance may be a good treatment option in some patients.

The present *in vitro* study is the first study evaluating the antifungal efficacy of nano-caffeic acid particles against candidiasis. Thus, the comparison and discussion of the results with other studies was impossible. However, these findings provide a strong foundation for further studies evaluating the antifungal effectiveness of this substance in *ex vivo* and *in vivo*. In this study, we mainly focused on evaluating the antifungal efficacy and physicochemical characteristics of caffeic acid and its nanoform. However, it is important to note that future studies should investigate the possible mechanisms involved in their antifungal activity. Based on previous findings, these mechanisms may include reactive oxygen species (ROS) generation, disruption of fungal cell membranes, and regulation of key genes such as *ERG11* and *HSP90*. Exploring these aspects would provide a better understanding of how these compounds work and support their potential clinical use.

## Conclusions

In the present study, span 60 and tween 60 were used along with cholesterol by ethanol injection to synthesize caffeic acid-containing niosomes. The results of this study showed that the optimal niosomal system contains caffeic acid with 42.34% loading, 271.83 nm size, −5.58 zeta potential, and 0.21 particle size distribution. In the present study, the highest to lowest MIC values ​​were observed for the pharmaceutical suspensions of caffeic acid, nano-caffeic acid, carrier, fluconazole, and nystatin against *Candida* species, respectively. Also, in this study, according to the MIC_50_ and MIC_90_ values, nystatin, fluconazole, the carrier, nano-caffeic acid, and caffeic acid had the greatest to lowest inhibitory properties against *Candida* species, respectively.

The present *in vitro* study provides a foundation for future *ex vivo* and *in vivo* studies evaluating the antifungal effectiveness of caffeic and nano-caffeic acid.

## Acknowledgements

We would like to thank the research center of Mazandaran University of Medical Sciences and all who helped in the completion of this study.

## Data Availability

The authors confirm that the data supporting the findings of this study are available within the article.
